# Fucoxanthin and Rosmarinic Acid Combination Has Anti-Inflammatory Effects through Regulation of NLRP3 Inflammasome in UVB-Exposed HaCaT Keratinocytes

**DOI:** 10.3390/md17080451

**Published:** 2019-08-01

**Authors:** Azahara Rodríguez-Luna, Javier Ávila-Román, Helena Oliveira, Virginia Motilva, Elena Talero

**Affiliations:** 1 Department of Pharmacology, Faculty of Pharmacy, Universidad de Sevilla, 41012 Seville, Spain; 2 Department of Biology, Faculty of Biology, University of Aveiro, 3810-193 Aveiro, Portugal

**Keywords:** fucoxanthin, rosmarinic acid, NRLP3, inflammasome, anti-oxidative, anti-inflammatory, photo-protection, UVB

## Abstract

Excessive exposure to ultraviolet (UV) radiation is the main risk factor to develop skin pathologies or cancer because it encourages oxidative condition and skin inflammation. In this sense, strategies for its prevention are currently being evaluated. Natural products such as carotenoids or polyphenols, which are abundant in the marine environment, have been used in the prevention of oxidative stress due to their demonstrated antioxidant activities. Nevertheless, the anti-inflammatory activity and its implication in photo-prevention have not been extensively studied. Thus, we aimed to evaluate the combination of fucoxanthin (FX) and rosmarinic acid (RA) on cell viability, apoptosis induction, inflammasome regulation, and anti-oxidative response activation in UVB-irradiated HaCaT keratinocytes. We demonstrated for the first time that the combination of FX and RA (5 µM RA plus 5 μM FX, designated as M2) improved antioxidant and anti-inflammatory profiles in comparison to compounds assayed individually, by reducing UVB-induced apoptosis and the consequent ROS production. Furthermore, the M2 combination modulated the inflammatory response through down-regulation of inflammasome components such as NLRP3, ASC, and Caspase-1, and the interleukin (IL)-1β production. In addition, Nrf2 and HO-1 antioxidant genes expression increased in UVB-exposed HaCaT cells pre-treated with M2. These results suggest that this combination of natural products exerts photo-protective effects by down-regulating NRLP3-inflammasome and increasing Nrf2 signalling pathway.

## 1. Introduction 

Skin is considered as the outmost protective barrier in the body, protecting from detrimental substances, mechanical damage, pathological invasion and radiation that could cause perturbations to the skin structure. In this sense, skin is a well-known essential piece of the immune system. Several factors can contribute to the initiation and development of cutaneous alterations. In this line, the excessive exposure to UV radiation remains the main risk factor for the skin cancer [1]. Solar UV radiation consists of three broad ranges of wavelength: UVC (100–280 nm), UVB (280–315 nm) and UVA (315–400 nm). UVB causes dermal changes, affects epidermal function and is the main factor responsible for the development of melanoma and non-melanoma skin cancer [2]. In this regard, a high dose of UVB exposure promotes cutaneous inflammation, which is traduced in sunburn, photo-aging, DNA damage, immunosuppression and induction of skin cancer [3]. 

It has been extensively studied that UVB-induced ROS production leads to activation of mitogen-activated protein kinase (MAPK) and nuclear factor-κB (NF-κB), among others, which further promote inflammation and apoptosis in cells and increase skin aging [4]. Furthermore, this type of inflammation results in the production of cytokines as tumour necrosis factor alpha (TNF-α), IL-6 and IL-1β, which are released by keratinocytes after UVB irradiation [5]. In this sense, it has been reported the relation between IL-1β secretion and activation of protein complexes called inflammasome [6]. Inflammasome is a wide cytosolic multiprotein complex that acts as mediator of the innate immune system, which is activated by multiple types of tissue damages. The nucleotide-binding domain, leucine-rich-repeat-containing family, pyrin domain-containing 3 (NLRP3) is the most studied inflammasome. NLRP3 gen induction results in activation of caspase-1, which by cleavage catalyses the processing of pro-IL-1β in cytosol to mature form, leading to IL-1β production in the extracellular medium [7]. In the last years, some authors have demonstrated the implication of NLRP3 inflammasome in tumorigenesis and cancer development, specifically in basal cell carcinomas [8,9].

Nowadays, non-melanoma skin cancer (NMSC) remains the most frequently diagnosed cancer in Caucasian people worldwide and strategies for its prevention are being developed [10]. At this regards, photo-chemoprevention by natural products is one of the most studied alternatives for skin protection, due to their anti-oxidative actions [1]. Although the anti-inflammatory properties of natural compounds have not been extensively investigated yet, in recent years, different studies have evaluated the beneficial effects of these compounds in photoprotection through their anti-inflammatory activity [1]. 

In last years, microalgae have been employed as a vast source of bioactive molecules with potential activity in inflammation and cancer [11]. Specifically, carotenoids have shown antioxidant, anti-inflammatory or anti-carcinogenic properties in several skin inflammatory models [12,13]. The orange carotenoid fucoxanthin (FX) is not exclusive from marine environmental [14] but it is an extensive compound produced by microalgae and brown seaweeds, as previously published [15] and it is known for its potent antioxidant properties. Nevertheless, its effect on NRLP3-inflammasome regulation has been little explored.

Polyphenols are the most popular antioxidant molecules in our diet, and exhibit other properties as anti-inflammatory or anti-neoplastic agents. For this reason, these compounds are also proposed as an important key for the management of skin protection [16]. In this regard, rosmarinic acid (RA) is a phenolic ester that has been traditionally isolated from some terrestrial plants as *Rosmarinus officinalis* L. or *Melissa officinalis* L. [17]. Moreover, this polyphenol has been abundantly found in *Zostera marina* seagrass beds [18,19]. This aspect is very interesting since *Zostera* has a rapid generation time and similar requirements to microalgae; thus it could be a great source to obtain a traditional phenolic compound like this. RA has been widely studied due to its remarkable biological and pharmacological activities, including anti-microbial, antioxidant and anti-inflammatory properties [20]. Until now, only a limited number of studies have dealt with the effect of RA on NLRP3-inflammasome [21,22,23], and none of them on human immortalized HaCaT keratinocytes. 

These antecedents led us to evaluate the effects of a combination of FX and RA on ROS production, apoptosis prevention, cell cycle alterations, inflammasome regulation and Nrf2 pathway activation, in UVB-exposed human keratinocytes.

## 2. Results 

### 2.1. Effects of RA and FX on Cell Viability 

**Figure 1:**
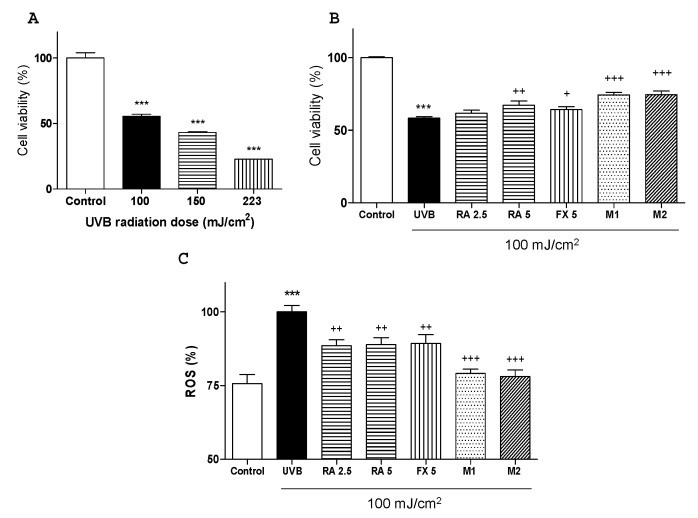
Selection of UVB dose and effect of rosmarinic acid (RA), fucoxanthin (FX) and combinations on cell viability and ROS production in UVB-irradiated HaCaT cells. (**A**) Effect of different UVB doses on cell viability determined by MTT assay. Results are expressed as percentage respect to untreated control cells and bars represents mean ± SEM of four independent experiments (*n* = 4) in duplicate. (*** *p* < 0.001 vs. untreated cells; Student t test) (**B**) Effect of RA, FX and their combinations on cell viability in human HaCaT keratinocytes was measured by MTT assay after 24 h of UVB exposition. (**C**) Intracellular ROS generation was evaluated 30 min after UVB irradiation by relative fluorescence intensity using 2’,7’-dichlorodihydrofluorescein diacetate (DCF-DA) assay. For experiments B and C, cells were pre-treated with RA (2.5 and 5 µM), FX (5 µM) and two combinations M1 (2.5 RA plus 5 µM FX) and M2 (5 µM RA plus 5 μM FX), for 1 h. Then, cells were irradiated with selected UVB dose (100 mJ/cm^2^) and incubated for the required times. Results are expressed as percentage respect to untreated control cells (**B**) or UVB-exposed control cells (**C**), and bars represents mean ± SEM of at least six independent experiments (*n* = 6) in duplicate. The mean was significantly different compared to control cells (*** *p* < 0.001; Student t test). The mean was significantly different compared to UVB-irradiated cells (^+^ *p* < 0.05, ^++^ *p* < 0.01 and ^+++^ *p* < 0.001; one-way ANOVA followed by Bonferroni’s multiple comparison test).

The UVB intensity was selected in a preliminary study in which three different intensities (100, 150 and 225 mJ/cm^2^) were tested. To study the cell viability was used MTT assay (3-(4,5-dimethylthiazol-2-yl)-2,5-diphenyltetrazolium bromide). As expected, a UVB dose-dependent effect on cell death and morphology was observed. The intensity of 100 mJ/cm^2^ was chosen for further assays since it allowed over 50% of cell survival and showed a significant difference in comparison with non-irradiated control group (Figure 1A). In addition, MTT results showed that the treatment with RA or FX at 5 µM, M1 (2.5 μM RA + 5 μM FX) and M2 (5 μM RA + 5 μM FX) significantly increased cell viability in UVB-exposed cells, being more effective M1 and M2 treatments (Figure 1B).

### 2.2. Effect of RA and FX on UVB-induced ROS Production. 

It is well known that intracellular ROS levels can be increased by UVB exposure [24]. In this sense, HaCaT cells were treated with the compounds for 1 h and then were irradiated with UVB (100 mJ/cm^2^). Cells exposed to UVB showed a significant increase in ROS production in comparison with non-exposed control cells (*p* < 0.001). The pre-treatment with either RA at the concentration of 2.5 or 5 μM or FX at 5 μM notably reduced intracellular ROS levels by 12%, 12% and 10%, respectively (*p* < 0.01). Nevertheless, concomitant administration of RA and FX (M1 or M2) showed a more marked decrease in ROS production, by 21% and 22%, respectively (*p* < 0.001) than the single treatments (Figure 1C).

### 2.3. Effect of RA and FX on Apoptosis

**Figure 2:**
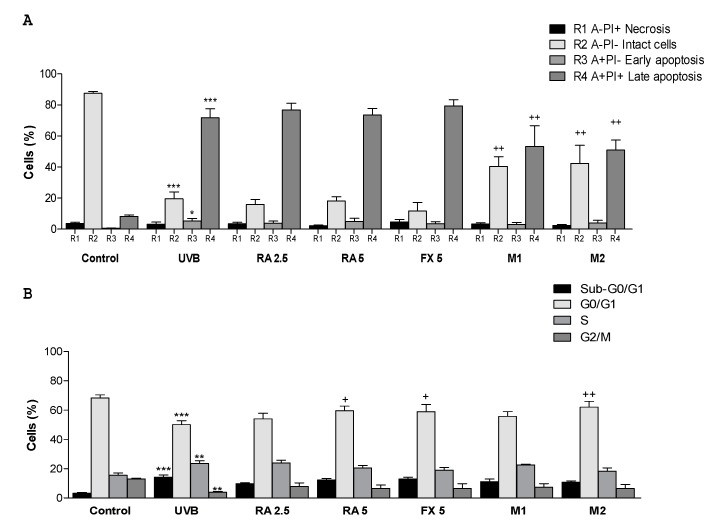
Effect of rosmarinic acid (RA), fucoxanthin (FX) and combinations on apoptosis and cell cycle arrest by using flow cytometry. (**A**) Results of Annexin V-FITC assay as percentage of cells in different apoptotic phases; R1: necrotic cells (Annexin V-FITC negative, PI positive), R2: viable cells (Annexin V-FITC negative, PI negative), R3: early apoptotic cells (Annexin V-FITC positive, PI negative), R4: late apoptotic cells (Annexin V-FITC positive, PI positive). Cells were pre-treated with RA (2.5 and 5 µM), FX (5 µM) and two combinations M1 (2.5 RA plus 5 µM FX) and M2 (5 µM RA plus 5 μM FX), then were irradiated with selected UVB dose (100 mJ/cm^2^) and incubated for 24 h. (**B**) Cell cycle phase distribution of UVB-exposed HaCaT cells. Cells were incubated with treatments for 48 h. Data are expressed as percentage and bars represents mean ± SEM of four independent experiments (*n* = 4) in duplicate. Results are expressed as percentage and bars represents mean ± SEM of four independent experiments (*n* = 4) in duplicate. The mean value was significantly different compared with control group (*** *p* < 0.001, ** *p* < 0.01, * *p* < 0.05 vs. untreated cells; Mann–Whitney U-test). The mean value was significantly different compared with UVB-irradiated cells ^+^ *p* < 0.05, ^++^ *p* < 0.01 vs. UVB-irradiated cells; Kruskal–Wallis test followed by Dunn’s multiple comparison test).

It is well reported that apoptosis is the most important type of programmed cell death in response to cell damage induced by UVB exposure [25]. In this sense, Annexin V (annexin family of intracellular protein)-FITC assay allows to differentiate cell subpopulations in different stages as necrotic (R1), viable (R2), early apoptosis (R3) or late apoptosis (R4). For this assay, only the adherent cells were considered, which means that the dead cells in suspension after UVB irradiation were not analysed and thus significant changes were not observed in this phase [26]. Our results showed that UVB exposure, in comparison with non-irradiated cells, significantly decreased the percentage of viable cells from 88% to 20%, increased the number of cells in early apoptosis from 1% to 5% and strongly augmented the percentage of cells in late apoptosis from 8% to 72% (Figure 2A). Regarding to pre-treatments, neither RA nor FX exhibited percentages changes in the different subpopulations, respect to UVB-exposed cells. Nevertheless, the treatment with M1 or M2 allowed a significant reduction of the percentage of late apoptotic cells and a marked increase of viable cells in comparison with irradiated control cells (Figure 2A).

### 2.4. Effect of RA and FX on Cell Cycle

It is well known that UV light exposure promotes skin photo-damage such as cell death and DNA damage and, consequently, cell cycle arrest [27]. Our results indicated that UVB-exposure highly affects the percentage of cells at G0/G1 phase, showing a reduction in this percentage from 68% to 50%, accompanied by an increase in apoptotic sub-G1 subpopulations from 3% to 14%, compared with non-exposed cells (Figure 2B). Moreover, UVB-exposed control group showed a significant increase in the percentage of cells in S phase, from 16% to 24%, and a marked decrease of cells in G2/M phase from 13% to 3% in comparison with non-irradiated control group. Pre-treatment with RA or FX at 5 µM, as well as concomitant administration of RA and FX at 5 µM (M2) significantly augmented the number of cells at G0/G1 phase in comparison with UVB-exposed cells. However, the percentage of cells in S phase was only reduced by M2, reaching similar values that those in control group (Figure 2B).

### 2.5. Effect of RA and FX on Inflammasome Regulation 

**Figure 3:**
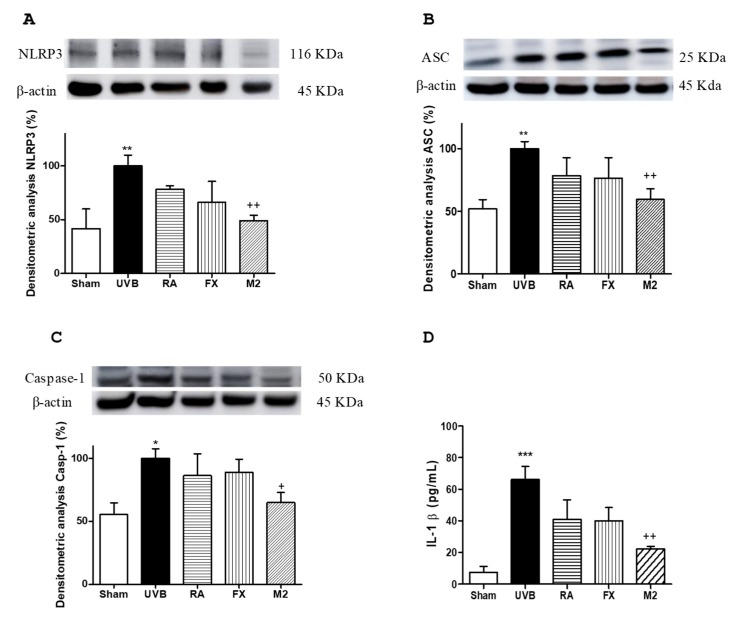
Effect of rosmarinic acid (RA), fucoxanthin (FX) and concomitant administration of RA and FX (M2) on inflammasome components expression and IL-1β levels in UVB-exposed HaCaT keratinocytes. Cells were pre-treated with RA (5 µM), FX (5 µM) and M2 (5 µM RA plus 5 μM FX) for 1h, then were UVB-irradiated (100 mJ/cm^2^) and incubated for 24 h. Densitometry analysis of (**A**) nucleotide-binding domain, leucine-rich-repeat-containing family, pyrin domain- containing 3 (NLRP3), (**B**) inflammasome adaptor protein (ASC) and (**C)** caspase-1 positivity were performed following normalization to the control (β-actin housekeeping gene). (**D**) IL-1β production was evaluated by ELISA assay. Results are expressed as percentage respect to UVB-irradiated control and bars represents mean ± SEM of four independent experiments (*n* = 4). The mean value was significantly different compared with control group (* *p* < 0.05, ** *p* < 0.01 and *** *p* < 0.001; Mann–Whitney U-test). The mean value was significantly different compared with UVB-irradiated cells (^+^ *p* < 0.05 and ^++^ *p* < 0.01; Kruskal–Wallis test followed by Dunn’s multiple comparison test).

It has been well documented that inflammasome can be activated by several factors, among them UVB exposure [8]. In the present study, we demonstrated that UVB irradiation led to up-regulation of NLRP3, ASC and caspase-1 expression and, consequently, promoted a significant increase in IL-1β production in comparison with non-exposed cells (Figure 3A–D). Pre-treatment with either RA or FX at 5 µM did not significantly modify the expression levels of inflammasome-related proteins. Nevertheless, combination of RA and FX at 5 µM (M2) significantly down-regulated NLRP3 and ASC expression levels (*p* < 0.01) as well as caspase-1 levels (*p* < 0.05) in comparison with UVB-exposed cells. Furthermore, pre-treatment with M2 reduced IL-1β production in a significant manner (*p* < 0.01) (Figure 3D).

### 2.6. Effect of RA and FX on Nrf-2 Antioxidant Signaling Pathway

**Figure 4:**
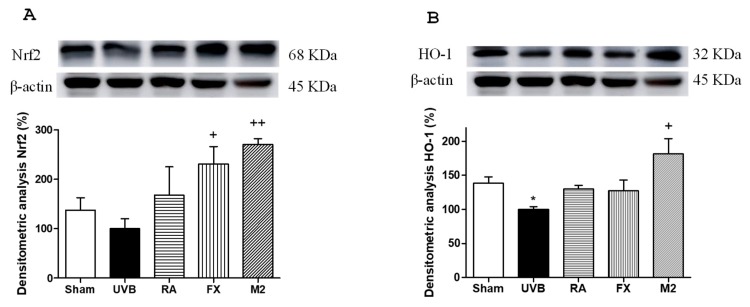
Effect of rosmarinic acid (RA), fucoxanthin (FX) and concomitant administration of RA and FX (M2) on Nrf2 and HO-1 expression in UVB-exposed HaCaT keratinocytes. Cells were pre-treated with RA (5 µM), FX (5 µM) and M2 (5 µM RA plus 5 μM FX) for 1 h, then were UVB-irradiated (100 mJ/cm^2^) and incubated for 24 h. Densitometry analysis of (**A**) Nrf2 and (**B**) HO-1 were performed following normalization to the control (β-actin housekeeping gene). Data shown are expressed as percentage respect to UVB-irradiated control and bars represents mean ± SEM of four independent experiments (*n* = 4). The mean value was significantly different compared with control group (* *p* < 0.05; Mann–Whitney U-test). The mean value was significantly different compared with UVB-irradiated cells (^+^ *p* < 0.05 and ^++^ *p* < 0.01; Kruskal–Wallis test followed by Dunn’s multiple comparison test.

To further elucidate the photo-protective mechanism of the combination of RA and FX, we evaluated the capacity to activate antioxidant pathways as Nrf2, which promotes the transcription of antioxidant genes and detoxification of enzymes such as heme-oxygenase 1 (HO-1) to protect against an oxidative damage as UVB exposure [28]. Our results showed that UVB irradiation prevented the increase of Nrf2 and HO-1 expression (Figure 4A–B). Non-irradiated control cells (sham) did not exhibit the highest expression levels of these antioxidant proteins because these cells were not exposed to any stress. Nevertheless, UVB-exposed HaCaT cells and pre-treated with FX at 5 µM or the combination of RA and FX at 5 µM (M2) significantly up-regulated Nrf2 expression levels (*p* < 0.05 and *p* < 0.01, respectively), reaching higher values that those in control cells. Regarding HO-1 expression, only M2 was able to significantly enhance this protein levels (*p* < 0.05).

## 3. Discussion 

Skin represents the first barrier that protects us from the deleterious effects of solar UV radiation, which is the main cause for skin cancer. Nevertheless, human skin is normally exposed to UV irradiation in a non-controlled way. Whereas a short-term UV exposure might suppress immune function or trigger an inflammatory response, chronic UV exposition conduces to photo-aging, DNA damage, or carcinoma [29]. Skin has an antioxidant system, composed by enzymatic and non-enzymatic antioxidants, to prevent against toxic exogenous/endogenous ROS levels. Nowadays, it is well known the importance of natural substances that support the endogenous antioxidant systems of the skin via diet or dermatological preparations [30]. To develop new skin photo-protective agents it is necessary to understand the molecular mechanism of UV-induced cellular responses and determine how these products may act in the skin cells. In this sense, many treatments with a unique compound have demonstrated successful results against photo-damage, but a more hopeful impact was obtained when a combination of several compounds was used [31]. The efficacy of combinations could be due to the synergistic effect of their components. In this regard, natural products may be good candidates for skin photo-protection due to their low toxicity and high antioxidant capacity [32]. Thus, the present study aimed to evaluate the preventive effect of a combination of RA and FX in UVB-exposed human HaCaT keratinocytes. 

It is well reported that UVB radiation increases ROS production and DNA damage as well as promotes a strong inflammatory response characterized by pro-inflammatory mediator production [33]. In this condition, apoptosis activation can eliminate the irreversibly damaged cells that could harbour oncogenic mutation [25]. In this line, FX has demonstrated antioxidant activity offering protection against DNA damage and preventing cellular apoptosis in HaCaT human keratinocytes [34,35]. In a similar way, several studies have reported that RA reduces ROS production, protects against DNA damage and regulates apoptotic markers in vitro [31,36]. According to these findings, our results showed that the pre-treatment with FX or RA at 5 µM significantly attenuated UVB-induced damage by increasing cell viability and inhibiting ROS production in human HaCaT keratinocytes, but did not modify the number of apoptotic cells after UVB exposure. Nevertheless, the selected combination of RA and FX at 5 µM at equal dose (M2) not only improved cell viability and ROS levels in comparison with FX and RA administered separately, but also enhanced the cellular protection, reducing the number of cells in apoptosis after UVB irradiation. In addition to cellular responses previously detailed, UVB exposure also affected cell cycle dynamics. Previous studies have reported that UV exposure activates cell cycle arrest in both G1 and G2 phases [37]. At this respect, our results reported that UVB irradiation significantly reduced cells at G0/G1 phase when compared with non-exposed cells. Furthermore, a S-phase delay was observed in UVB-exposed cells although it did not show to be highly affected by the treatments. The pre-treatment with FX, RA and concomitant administration of RA and FX (M2) significantly increased the percentage of cells at G0/G1 and reduced the percentage of cells at sub-G1. In this sense, our observations are in accordance with the results obtained in apoptosis assay. M2 prevented cell damage, reducing the percentage of cells under apoptosis 24 h after UVB exposure. In consequence, this combination promoted a higher percentage of viable cells at 48 h in UVB-exposed cells.

In terms of inflammation, it has been reported that pro-IL-1β is cleaved and IL-1β is released from cells as a pro-inflammatory cytokine after NLRP3 inflammasome activation [38]. Thus, it could be concluded that IL-1β production in keratinocytes is inflammasome-dependent [6]. Moreover, NLRP3 inflammasome activation by UVB increases not only IL-1β but also other pro-inflammatory cytokines such as IL-1α, IL-6, TNF-α or the mediator PGE2 [7]. In this line, NLRP3 inflammasome plays an important role in UVB-induced skin inflammatory responses and has a critical function in skin pathologies initiation such as cancer [8]. In addition, the inflammasome adaptor protein ASC has shown anti-tumour activity by activation of tumour suppressor protein p53 in UVB-irradiated keratinocytes [39]. Due to the high connection between inflammasome, inflammation and cancer, the therapeutic strategies targeting inflammasome-related products are currently in development and suppose a pivotal tool for skin pathologies and cancer prevention [40]. In previous studies, FX has shown anti-inflammatory activity through NF-κB and MAPKs signalling pathways regulation [41], and consequently the down-regulation of IL-1β, TNF-α, inducible nitric oxide synthase, and cyclooxygenase-2 expression [42,43,44]. On the other hand, the polyphenol RA has been previously reported to inhibit pro-inflammatory cytokines production, including IL-1β, and to down-regulate NF-κB signalling pathway in skin cells [45]. In addition, this polyphenol has shown to modulate inflammasome activation, through down-regulation of caspase-1, NLRP3 and ASC expression in a poly(I:C)-induced inflammation in an infectious model by using epidermal keratinocytes [21]. Moreover, due to its anti-inflammatory properties, RA has been proposed to ameliorate allergic reactions in allergic rhinitis and rhinoconjunctivitis [46]. Furthermore, this compound has shown to prevent cisplatin-induced apoptosis in chemotherapy by down-regulating the caspase-1 expression [47]. However, the role of FX or RA on NLRP3 inflammasome regulation in human keratinocytes has not been studied yet. In this line, our findings reported for the first time that the combination of RA and FX notably reduced inflammasome-related proteins such as NLRP3, ASC and caspase-1 in UVB-irradiated HaCaT keratinocytes. Interestingly, this effect was not detected when both compounds were administered separately, suggesting the beneficial effect of concomitant treatment with RA and FX.

The antioxidant role of FX and RA has been well-described. FX has shown to promote AKT/Nrf2/GSH-dependent antioxidant response in keratinocytes [48,49], as well as reduce wrinkles formation and epidermal hypertrophy in mice [50] and supress melanogenesis and prostaglandin synthesis [51]. In this line, due to its antioxidant activity, this carotenoid has been proposed as a photo-protective compound by stimulating skin barrier restoration in UVA-induced sunburn [52]. Furthermore, a previous study by our group evidenced that FX increased Nrf2 and HO-1 expression in UVB-induced acute erythema in SKH-1 hairless mice [44]. These results are in line with other authors that suggest that this compound could be an interesting strategy to counteract UVB-induced oxidative damage in skin [53]. At this regard, similar properties have been demonstrated with RA applications, confirming the antioxidant activity of this polyphenol via activation of Nrf2-antioxidant system [54,55]. In accordance with these findings, the combination between FX and RA (M2) used in this study showed an up-regulation of Nrf2 transcriptional factor and its main target gene HO-1. These data indicate that the protective activity of M2 may be due to the reduction of oxidative stress through modulation of Nrf2 signalling pathway.

## 4. Materials and Methods

### 4.1. Compounds 

RA and FX were obtained from Sigma-Aldrich (St. Louis, MO, USA). RA and FX stocks were prepared in DMSO at 10 mM and diluted to the desired concentration with culture medium. Controls were incubated with the corresponding quantity of DMSO, which was always below 1% and did not affect cell viability

### 4.2. Cell Line

Human immortalized keratinocytes HaCaT were obtained from the American Type Culture Collection (ATCC) and maintained in high glucose Dulbecco’s modified Eagle’s medium (DMEM, GIBCO, Grand Island, NY, USA) containing 2 mM l-glutamine. The culture media was supplemented with 10% heat-inactivated fetal bovine serum, 100 U/mL penicillin and 100 mg/mL streptomycin (PAA Laboratories, Pasching, Austria). Cultures were incubated in an atmosphere of 5% CO_2_ at 37 °C.

### 4.3. UVB-Irradiation 

The CL-1000M UV Crosslinker system (UVP, Upland, CA, USA), formed by 5 UVB tubes (8 W), was used to submit an energy spectrum of UVB radiation (peak intensity 302 nm, inside of the spectrum of UVB light 280–315 nm). To prevent UVB light absorption by the cell culture medium the medium was replaced by a thin layer of phosphate buffer solution (PBS) to cover the cells during irradiation. Three UVB intensities were evaluated (100, 150 and 225 mJ/cm^2^) to determine the most suitable to evaluate cell cycle alterations and apoptosis [22]. In this sense, 100 mJ/cm^2^ was selected for the study.

### 4.4. Cell Proliferation Assay

Cell viability was evaluated by the colorimetric 3-(4,5-dimethyl-2-thiazolyl)-2,5-diphenyl tetrazolium bromide (MTT) (Sigma-Aldrich, St. Louis, MO, USA) assay, which determines the formation of purple formazan in viable cells and allows estimate cellular viability [56]. MTT assay was performed according to protocol described with slight modifications. Briefly, HaCaT cells were seeded at a density of 10^4^ cells/well in a 96-well plate for 24 h. Cells were pre-treated with different doses either RA (2.5, 5, 7.5 and 10 µM) or FX (5, 10, 15 and 20 µM) and the sixteen possible combinations between RA and FX, for 1 h. Then, the culture medium was replaced with a thin layer of PBS and exposed to a single dose of UVB radiation at 100 mJ/cm^2^. Cells were supplied with fresh culture medium and incubated for 24 h. Absorbance was measured at 570 nm using a Synergy HT Multi-mode Microplate Reader (BioTek Instruments, Winooski, VT, USA). 

### 4.5. Intracellular ROS Scavenging Activity

The 2’,7’-dichlorodihydrofluorescein diacetate (DCF-DA) assay was used to detect intracellular ROS levels in HaCaT keratinocytes [57]. Briefly, keratinocytes were seeded at 10^4^ cells/well in 96-well plates and were treated with RA (2.5 and 5 µM), FX (5 µM) and the combinations M1 (2.5 RA plus 5 µM FX) and M2 (5 µM RA plus 5 μM FX) for 1 h. After UVB exposure (100 mJ/cm^2^), cells were incubated with 2’,7’-dichlorodihydrofluorescein diacetate (DCFH-DA) solution (5 mg/mL) in PBS for 30 min at 37 °C. Then, the medium was discarded and the cells were washed with PBS. The fluorescence of the 2’,7’-dichlorofluorescein (DCF) product was determined by using a fluorescence plate reader (Sinergy HT, Biotek^®^, Bad Friedrichshall, Germany) at 485 nm for excitation and 535 nm for emission.

### 4.6. Apoptosis Determination

Apoptosis was evaluated by flow cytometry using Annexin V-FITC Apoptosis detection kit from BD Pharmingen (Franklin Lakes, NJ, USA), as previously reported [26]. Briefly, HaCaT cells were seeded at 5 × 10^5^ cells/well in 6-well plates and incubated for complete adhesion. The day after seeding, cells were treated with RA (2.5 and 5 µM), FX (5 µM) and mixtures M1 (2.5 RA plus 5 µM FX) and M2 (5 µM RA plus 5 μM FX) for 1 h. After that, treatments were removed and cells were irradiated at 100 mJ/cm^2^, and then incubated with fresh medium for 24 h. Cells were harvested and suspended in binding buffer at 10^5^ cells/mL. Then, annexin V-FITC and propidium iodide (PI) were added as indicated in the manufacture’s protocol. Subsequently, samples were kept in darkness for 15 min and 400 µL of binding buffed were added. Fluorescence intensity of PI and FITC-Annexin-V-stained cells was determined on a Coulter Epics XL Flow Cytometer (Beckman Coulter, Hialeah, FL, USA). Data were obtained using the SYSTEM II (v.2.5.) software examining 10^4^ events. The cytogram of FITC fluorescence in log scale versus PI fluorescence in log scales allows the identification of viable cells (Annexin V-FITC negative, PI negative), early apoptotic cells (Annexin V-FITC positive, PI negative), late apoptotic cells (Annexin V-FITC positive, PI positive) and necrotic cells (Annexin V-FITC negative, PI positive). Flow cytometry data were analysed by FlowJo software (Tree Star Inc., Ashland, OR, USA).

### 4.7. Cell Cycle Determination 

For cell cycle determination, a similar procedure to apoptosis evaluation was carried out, with the difference of that after irradiation, cells were incubated with fresh medium for 48 h. After incubation, keratinocytes were harvested by trypsinization as previously detailed [26], fixed in 85% cold ethanol (5 × 10^5^ cells/mL) and kept at −20 °C until further analysis. Ethanol was eliminated, cells were resuspended in PBS, and the cell suspension was filtered through a 35 µm nylon mesh to separate aggregated cells. Then, 50 μL RNase (1 mg/mL) and 50 μL PI (1 mg/mL) were added to each sample, which was then incubated for 20 min in darkness at room temperature until analysis. DNA content was determined on a Coulter Epics XL Flow Cytometer (Beckman Coulter, Hialeah, FL, USA). Data were acquired using the SYSTEM II (v. 2.5.) software examining 10^4^ events. Percentages of cells in apoptotic-sub G1, G0/G1, S and G2/M were calculated using CXP software.

### 4.8. Determination of IL-1β Production

HaCaT cells were seeded at 5 × 10^5^ cells/well in 6-well plates, incubated for 24 h and treated with either RA (5 µM), FX (5 µM) or M2 (5 µM RA plus 5 μM FX), for 1 h. Then, the cells were irradiated at 100 mJ/cm^2^, and incubated with fresh medium for 24 h. Supernatant fluids were collected and stored at −80 °C until use. Commercial enzyme-linked immunosorbent assay (ELISA) kits (Diaclone GEN-PROBE, Besançon cedex, France) was used to quantify IL-1β production according to the manufacturer’s protocol. The absorbance at 450 nm was read by a microplate reader (Sinergy HT, Biotek^®^, Bad Friedrichshall, Germany). To calculate the concentration of IL-1β (pg/mL), a standard curve was constructed using serial dilutions of cytokine standards provided with the kit.

### 4.9. Western Blot Analysis

The cell culture and treatments were similar to those carried out for cytokine production. Then, HaCaT cells were harvested by trypsinization, centrifuged and resuspended in lysis buffer (50 mM HEPES, 150 mM NaCl, 1 mM EDTA, 1 mM EGTA, 10% glycerol, 1% Triton-X-100, 1 mM phenylmethylsulphonyl fluoride, protease inhibitor cocktail tablet, 0.5 mM sodium orthovanadate, 20 mM sodium pyrophosphate). The homogenates were centrifuged (12,000 g, 15 min, 4 °C), and the supernatants were collected and stored at −80 °C. Bradford colorimetric method was utilized to determine the protein concentration of the homogenates [58]. Samples of the supernatants with equal amounts of protein (20 μg) were separated on 10% acrylamide gel by sodium dodecyl sulphate polyacrylamide gel electrophoresis. Next, the proteins were electrophoretically transferred onto a nitrocellulose membrane and incubated with specific primary antibodies: rabbit anti-ASC (Bio-Rad, Hercules, CA, USA) (1:1000), rabbit anti-NLRP3 (1:1000), rabbit anti-Nrf2 (1:1000), rabbit anti-HO-1 (1:1000), rabbit anti-caspase-1 (1:1000) (Cell Signaling, Danvers, MA, USA) overnight at 4 °C. After rising, the membranes were incubated with the horseradish peroxidase-linked (HRP) secondary antibody anti-rabbit (Cayman Chemical^®^, Ann Arbor, MI, USA) (1:1000) or anti-mouse (Dako^®^, Atlanta, GA, USA) (1:1000) containing blocking solution for 1 h at room temperature. To prove equal loading, the blots were analysed for β-actin expression using an anti-β-actin antibody (Sigma Aldrich^®^, St. Louis, MO, USA). Immunodetection was performed employing an enhanced chemiluminescence light-detecting kit (SuperSignal West Pico Chemiluminescent Substrate, Pierce, IL, USA). Then, the immunosignals were monitored by using an Amersham Imaging 600 (GE Healthcare Life Sciences, Barcelona, Spain) and densitometric data were analysed after normalization to the control (housekeeping gene). The signals were analysed and quantified with a Scientific Imaging Systems (Biophotonics ImageJ Analysis Software, National Institute of Mental Health, Bethesda, MD, USA) and expressed as total percentage respect to UVB-exposed control group.

### 4.10. Statistical Analysis

All values in the figures and text are expressed as arithmetic means ± standard error of the mean (S.E.M.). Data were evaluated with GraphPad Prism^®^ Version 5.00 software (San Diego, CA, USA). In all cases, the Shapiro–Wilk test was used to verify the normality of the data. The Mann–Whitney U-test was chosen for non-parametric values. The parametric values groups were analysed by one-way analysis of variance (ANOVA) followed by Bonferroni’s multiple comparison test. *p* values < 0.05 were considered statistically significant.

## 5. Conclusions

In conclusion, we have demonstrated for the first time that the combination of RA and FX shows additional benefits in comparison with the compounds administered separately on UVB-irradiated keratinocytes. The studied combination (M2) increased cell viability by reducing UVB-induced apoptosis and decreased intracellular ROS production. Moreover, the concomitant administration of RA and FX showed anti-inflammatory activity through NLRP3 inflammasome modulation, as well as antioxidant properties via up-regulation of Nrf2/HO-1 antioxidant system. Overall, we propose the combination of RA and FX as a natural promising tool in prevention of UVB-induced skin alterations as photo-aging, skin inflammation and its derivation to pre-cancerous lesions and skin carcinomas.

## Abbreviations

ASC	apoptosis-attached-speck-like protein containing a CARD
DCFH-DA	2’,7’-dichlorodihydrofluorescein diacetate
FX	Fucoxanthin
HO-1	heme oxygenase 1
IL	Interleukin
NMSC	non-melanoma skin cancer
NLRP3	nucleotide-binding domain, leucine-rich-repeat-containing family, pyrin domain- containing 3
Nrf2	nuclear factor E2-related factor 2
ROS	reactive oxygen species
SRB	sulforhodamine B
TNF-α	tumour necrosis factor alpha
TPA	12-O-tetradecanoylphorbol-13-acetate
UV	ultraviolet

## References

[1] Alam S., Pal A., Singh D., Ansari K.M. (2018). Topical Application of Nexrutine Inhibits UVB-induced Cutaneous Inflammatory Responses in SKH-1 Hairless Mouse. Photodermatol. Photoimmunol. Photomed..

[2] Katiyar S.K., Pal H.C., Prasad R. (2017). Dietary Proanthocyanidins Prevent Ultraviolet Radiation-Induced Non-Melanoma Skin Cancer Through Enhanced Repair of Damaged DNA-Dependent Activation of Immune Sensitivity. Semin. Cancer Biol..

[3] Hatakeyama M., Fukunaga A., Washio K., Taguchi K., Oda Y., Ogura K., Nishigori C. (2017). Anti-Inflammatory Role of Langerhans Cells and Apoptotic Keratinocytes in Ultraviolet-B-Induced Cutaneous Inflammation. J. Immunol..

[4] Subedi L., Lee T.H., Wahedi H.M., Baek S.H., Kim S.Y. (2017). Resveratrol-Enriched Rice Attenuates UVB-ROS-Induced Skin Aging Via Downregulation of Inflammatory Cascades. Oxid. Med. Cell. Longev..

[5] Kondo S., Sauder D.N., Kono T., Galley K.A., McKenzie R.C. (1994). Differential Modulation of Interleukin-1 Alpha (IL-1α) and Interleukin-1 beta (IL-1β) in Human Epidermal Keratinocytes by UVB. Exp. Dermatol..

[6] Feldmeyer L., Keller M., Niklaus G., Hohl D., Werner S., Beer H.D. (2007). The Inflammasome Mediates UVB-Induced Activation and Secretion of Interleukin-1β by Keratinocytes. Curr. Biol..

[7] Hasegawa T., Nakashima M., Suzuki Y. (2016). Nuclear DNA Damage-Triggered NLRP3 Inflammasome Activation Promotes UVB-Induced Inflammatory Responses in Human Keratinocytes. Biochem. Biophys. Res. Commun..

[8] Ahmad I., Muneer K.M., Chang M.E., Nasr H.M., Clay J.M., Huang C.C., Yusuf N. (2017). Ultraviolet Radiation-Induced Downregulation of SERCA2 Mediates Activation of NLRP3 Inflammasome in Basal Cell Carcinoma. Photochem. Photobiol..

[9] Huang C.F., Chen L., Li Y.C., Wu L., Yu G.T., Zhang W.F., Sun Z.J. (2017). NLRP3 Inflammasome Activation Promotes Inflammation-Induced Carcinogenesis in Head and Neck Squamous Cell Carcinoma. J. Exp. Clin. Cancer Res..

[10] Zink A., Koch E., Seifert F., Rotter M., Spinner C.D., Biedermann T. (2016). Non-Melanoma Skin Cancer in Mountain Guides: High Prevalence and Lack of Awareness Warrant Development of Evidence-Based Prevention Tools. Swiss Med. Wkly..

[11] Talero E., García-mauriño S., Ávila-román J., Rodríguez-Luna A., Alcaide A., Motilva V. (2015). Bioactive Compounds Isolated from Microalgae in Chronic Inflammation and Cancer. Mar. Drugs.

[12] Oh J., Kim J.H., Park J.G., Yi Y.S., Park K.W., Rho H.S., Lee M.S., Yoo J.W., Kang S.H., Hong Y.D. (2013). Radical Scavenging Activity-Based and AP-1-Targeted Anti-Inflammatory Effects of Lutein in Macrophage-Like and Skin Keratinocytic Cells. Mediat. Inflamm..

[13] Yoshihisa Y., Andoh T., Matsunaga K., Rehman M.U., Maoka T., Shimizu T. (2016). Efficacy of Astaxanthin for the Treatment of Atopic Dermatitis in a Murine Model. PLoS ONE.

[14] Amicucci A., Barbieri E., Sparvoli V., Gioacchini A.M., Calcabrini C., Palma F., Stocchi V., Zambonelli A. (2018). Microbial and Pigment Profile of the Reddish Patch Occurring within Tuber Magnatum Ascomata. Fungal Biol..

[15] D’Orazio N., Gemello E., Gammone M.A., de Girolamo M., Ficoneri C., Riccioni G. (2012). Fucoxantin: A Treasure from the Sea. Mar. Drugs.

[16] Saric S., Sivamani R. (2016). Polyphenols and Sunburn. Int. J. Mol. Sci..

[17] Petersen M. (2013). Rosmarinic Acid: New Aspects. Phytochem. Rev..

[18] Wang J., Pan X., Han Y., Guo D., Guo Q., Li R. (2012). Rosmarinic Acid from Eelgrass Shows Nematicidal and Antibacterial Activities Against Pine Wood Nematode and Its Carrying Bacteria. Mar. Drugs.

[19] Guan C., Parrot D., Wiese J., Sönnichsen F.D., Saha M., Tasdemir D., Weinberger F. (2017). Identification of Rosmarinic Acid and Sulfated Flavonoids as Inhibitors of Microfouling on the Surface of Eelgrass *Zostera* Marina. Biofouling.

[20] Amoah S.K.S., Sandjo L.P., Kratz J.M., Biavatti M.W. (2016). Rosmarinic Acid-Pharmaceutical and Clinical Aspects. Planta Med..

[21] Zhou M.W., Jiang R.H., Kim K.D., Lee J.H., Kim C.D., Yin W.T., Lee J.H. (2016). Rosmarinic Acid Inhibits Poly(I:C)-Induced Inflammatory Reaction of Epidermal Keratinocytes. Life Sci..

[22] Wei Y., Chen J., Hu Y., Lu W., Zhang X., Wang R., Chu K. (2018). Rosmarinic Acid Mitigates Lipopolysaccharide-Induced Neuroinflammatory Responses through the Inhibition of TLR4 and CD14 Expression and NF-κB and NLRP3 Inflammasome Activation. Inflammation.

[23] Yao Y., Mao J., Xu S., Zhao L., Long L., Chen L., Li D., Lu S. (2019). Rosmarinic Acid Inhibits Nicotine-Induced C-Reactive Protein Generation by Inhibiting NLRP3 Inflammasome Activation in Smooth Muscle Cells. J. Cell. Physiol..

[24] Kovacs D., Raffa S., Flori E., Aspite N., Briganti S., Cardinali G., Torrisi M.R., Picardo M. (2009). Keratinocyte Growth Factor Down-Regulates Intracellular ROS Production Induced by UVB. J. Dermatol. Sci..

[25] Feehan R.P., Shantz L.M. (2016). Molecular Signaling Cascades Involved in Nonmelanoma Skin Carcinogenesis. Biochem. J..

[26] Ascenso A., Pedrosa T., Pinho S., Pinho F., de Oliveira J.M., Cabral-Marques H., Oliveira H., Simões S., Santos C. (2016). The Effect of Lycopene Preexposure on UV-B-Irradiated Human Keratinocytes. Oxid. Med. Cell. Longev..

[27] Lee J.J., Kim K.B., Heo J., Cho D.H., Kim H.S., Han S.H., Ahn K.J., An I.S., An S., Bae S. (2017). Protective Effect of *Arthrospira platensis* Extracts Against Ultraviolet B-Induced Cellular Senescence Through Inhibition of DNA Damage and Matrix Metalloproteinase-1 Expression in Human Dermal Fibroblasts. J. Photochem. Photobiol. B Biol..

[28] Furue M., Uchi H., Mitoma C., Hashimoto-Hachiya A., Chiba T., Ito T., Nakahara T., Tsuji G. (2017). Antioxidants for Healthy Skin: The Emerging Role of Aryl Hydrocarbon Receptors and Nuclear Factor-Erythroid 2-Related Factor-2. Nutrients.

[29] Sajo M.E.J., Kim C.S., Kim S.K., Shim K.Y., Kang T.Y., Lee K.J. (2017). Antioxidant and Anti-Inflammatory Effects of Shungite Against Ultraviolet B Irradiation-Induced Skin Damage in Hairless Mice. Oxid. Med. Cell. Longev..

[30] Chen L., Hu J.Y., Wang S.Q. (2012). The Role of Antioxidants in Photoprotection: A Critical Review. J. Am. Acad. Dermatol..

[31] Psotova J., Svobodova A., Kolarova H., Walterova D. (2006). Photoprotective Properties of Prunella vulgaris and Rosmarinic Acid on Human Keratinocytes. J. Photochem. Photobiol. B Biol..

[32] Dreher F., Gabard B., Schwindt D., Maibach H.I. (1998). Topical Melatonin in Combination with Vitamins E and C Protects Skin from Ultraviolet-Induced Erythema: A Human Study In Vivo. Br. J. Dermatol..

[33] Leerach N., Yakaew S., Phimnuan P., Soimee W., Nakyai W., Luangbudnark W., Viyoch J. (2017). Effect of Thai Banana (Musa AA group) in Reducing Accumulation of Oxidation End Products in UVB-Irradiated Mouse Skin. J. Photochem. Photobiol. B Biol..

[34] Heo S.J., Jeon Y.J. (2009). Protective Effect of Fucoxanthin Isolated from Sargassum Siliquastrum on UV-B Induced Cell Damage. J. Photochem. Photobiol. B Biol..

[35] Zheng J., Piao M.J., Keum Y.S., Kim H.S., Hyun J.W. (2013). Fucoxanthin Protects Cultured Human Keratinocytes Against Oxidative Stress by Blocking free Radicals and Inhibiting Apoptosis. Biomol. Ther. (Seoul).

[36] Vostálová J., Zdařilová A., Svobodová A. (2010). Prunella Vulgaris Extract and Rosmarinic Acid Prevent UVB-Induced DNA Damage and Oxidative Stress in HaCaT Keratinocytes. Arch. Dermatol. Res..

[37] Pavey S., Russell T., Gabrielli B. (2001). G2 Phase Cell Cycle Arrest in Human Skin Following UV Irradiation. Oncogene.

[38] Jang Y., Lee A.Y., Jeong S.H., Park K.H., Paik M.K., Cho N.J., Kim J.E., Cho M.H. (2015). Chlorpyrifos Induces NLRP3 Inflammasome and Pyroptosis/Apoptosis Via Mitochondrial Oxidative Stress in Human Keratinocyte HaCaT Cells. Toxicology.

[39] Drexler S.K., Bonsignore L., Masin M., Tardivel A., Jackstadt R., Hermeking H. (2012). Tissue-Specific Opposing Functions of the Inflammasome Adaptor ASC in the Regulation of Epithelial Skin Carcinogenesis. Proc. Natl. Acad. Sci. USA.

[40] Lin C., Zhang J. (2017). Inflammasomes in Inflammation-Induced Cancer. Front. Immunol..

[41] Choi J.H., Kim N.H., Kim S.J., Lee H.J., Kim S. (2016). Fucoxanthin Inhibits the Inflammation Response in Paw Edema Model Through Suppressing MAPKs, Akt, and NFκB. J. Biochem. Mol. Toxicol..

[42] Tan C., Hou Y. (2014). First Evidence for the Anti-Inflammatory Activity of Fucoxanthin in High-Fat-Diet-Induced Obesity in Mice and the Antioxidant Functions in PC12 Cells. Inflammation.

[43] Gong D., Chu W., Jiang L., Geng C., Li J., Ishikawa N., Kajima K., Zhong L. (2014). Effect of Fucoxanthin Alone and in Combination with D-Glucosamine Hydrochloride on Carrageenan/Kaolin-Induced Experimental Arthritis in Rats. Phyther. Res..

[44] Rodríguez-Luna A., Ávila-Román J., González-Rodríguez M.L., Cózar M.J., Rabasco A.M., Motilva V., Talero E. (2018). Fucoxanthin-Containing Cream Prevents Epidermal Hyperplasia and UVB-Induced Skin Erythema in Mice. Mar. Drugs.

[45] Lee J., Jung E., Kim Y., Lee J., Park J., Hong S., Hyun C.G., Park D., Kim Y.S. (2006). Rosmarinic Acid as a Downstream Inhibitor of IKK-Beta in TNF-Alpha-Induced Upregulation of CCL11 and CCR3. Br. J. Pharmacol..

[46] Oh H.A., Park C.S., Ahn H.J., Park Y.S., Kim H.M. (2011). Effect of Perilla Frutescens Var. Acuta Kudo and Rosmarinic Acid on Allergic Inflammatory Reactions. Exp. Biol. Med. (Maywood).

[47] Jeong H.J., Choi Y., Kim M.H., Kang I.C., Lee J.H., Park C., Park R., Kim H.M. (2011). Rosmarinic Acid, Active Component of Dansam-Eum Attenuates Ototoxicity of Cochlear Hair Cells Through Blockage of Caspase-1 Activity. PLoS ONE.

[48] Zheng J., Piao M.J., Kim K.C., Yao C.W., Cha J.W., Hyun J.W. (2014). Fucoxanthin Enhances the Level of Reduced Glutathione Via the Nrf2-Mediated Pathway in Human Keratinocytes. Mar. Drugs.

[49] Liu Y., Zheng J., Zhang Y., Wang Z., Yang Y., Bai M., Dai Y. (2016). Fucoxanthin Activates Apoptosis Via Inhibition of PI3K/Akt/mTOR Pathway and Suppresses Invasion and Migration by Restriction of p38-MMP-2/9 Pathway in Human Glioblastoma Cells. Neurochem. Res..

[50] Urikura I., Sugawara T., Hirata T. (2011). Protective Effect of Fucoxanthin Against UVB-Induced Skin Photoaging in Hairless Mice. Biosci. Biotechnol. Biochem..

[51] Shimoda H., Tanaka J., Shan S.J., Maoka T. (2010). Anti-Pigmentary Activity of Fucoxanthin and Its Influence on Skin mRNA Expression of Melanogenic Molecules. J. Pharm. Pharmacol..

[52] Matsui M., Tanaka K., Higashiguchi N., Okawa H., Yamada Y., Tanaka K., Taira S., Aoyama T., Takanishi M., Natsume C. (2016). Protective and Therapeutic Effects of Fucoxanthin Against Sunburn Caused by UV Irradiation. J. Pharmacol. Sci..

[53] Sun Z., Park S.Y., Hwang E., Park B., Seo S.A., Cho J.G., Zhang M., Yi T.H. (2016). Dietary Foeniculum Vulgare Mill Extract Attenuated UVB Irradiation-Induced Skin Photoaging by Activating of Nrf2 and Inhibiting MAPK Pathways. Phytomedicine.

[54] Sun Z., Park S.Y., Hwang E., Zhang M., Seo S.A., Lin P., Yi T.H. (2016). Thymus Vulgaris Alleviates UVB Irradiation Induced Skin Damage Via Inhibition of MAPK/AP-1 and Activation of Nrf2-ARE Antioxidant System. J. Cell. Mol. Med..

[55] Lu C., Zou Y., Liu Y., Niu Y. (2017). Rosmarinic Acid Counteracts Activation of Hepatic Stellate Cells Via Inhibiting the ROS-Dependent MMP-2 Activity: Involvement of Nrf2 Antioxidant System. Toxicol. Appl. Pharmacol..

[56] Twentyman P.R., Luscombe M. (1987). A Study of Some Variables in a Tetrazolium Dye (MTT) Based Assay for Cell Growth and Chemosensitivity. Br. J. Cancer.

[57] Wang H., Joseph J.A. (1999). Quantifying Cellular Oxidative Stress by Dichlorofluorescein Assay Using Microplate Reader. Free Radic. Biol. Med..

[58] Bradford M. (1976). A Rapid and Sensitive Method for The Quantitation of Microgram Quantities of Protein Utilizing the Principle of Protein-Dye Binding. Anal. Biochem..

